# Identification of Tumor Microenvironment-Related Alternative Splicing Events to Predict the Prognosis of Endometrial Cancer

**DOI:** 10.3389/fonc.2021.645912

**Published:** 2021-04-29

**Authors:** Xuan Liu, Chuan Liu, Jie Liu, Ying Song, Shanshan Wang, Miaoqing Wu, Shanshan Yu, Luya Cai

**Affiliations:** ^1^ Department of Obstetrics and Gynecology, Jinhua People’s Hospital, Jinhua, China; ^2^ Department of Medical Oncology, The First Hospital of China Medical University, Shenyang, China; ^3^ Department of Gynecology, Jinhua People’s Hospital, Jinhua, China; ^4^ Department of Chemoradiation Oncology, The First Affiliated Hospital of Wenzhou Medical University, Wenzhou, China; ^5^ Department of Obstetrics and Gynecology, The First Affiliated Hospital of Wenzhou Medical University, Wenzhou, China

**Keywords:** endometrial cancer, alternative splicing, tumor microenvironment, prognosis, gene signature

## Abstract

**Background:**

Endometrial cancer (EC) is one of the most common female malignant tumors. The immunity is believed to be associated with EC patients’ survival, and growing studies have shown that aberrant alternative splicing (AS) might contribute to the progression of cancers.

**Methods:**

We downloaded the clinical information and mRNA expression profiles of 542 tumor tissues and 23 normal tissues from The Cancer Genome Atlas (TCGA) database. ESTIMATE algorithm was carried out on each EC sample, and the OS-related different expressed AS (DEAS) events were identified by comparing the high and low stromal/immune scores groups. Next, we constructed a risk score model to predict the prognosis of EC patients. Finally, we used unsupervised cluster analysis to compare the relationship between prognosis and tumor immune microenvironment.

**Results:**

The prognostic risk score model was constructed based on 16 OS-related DEAS events finally identified, and then we found that compared with high-risk group the OS in the low-risk group was notably better. Furthermore, according to the results of unsupervised cluster analysis, we found that the better the prognosis, the higher the patient’s ESTIMATE score and the higher the infiltration of immune cells.

**Conclusions:**

We used bioinformatics to construct a gene signature to predict the prognosis of patients with EC. The gene signature was combined with tumor microenvironment (TME) and AS events, which allowed a deeper understanding of the immune status of EC patients, and also provided new insights for clinical patients with EC.

## Introduction

Endometrial cancer (EC) is one of the most common malignances in females (1), and it often occurs in perimenopausal and postmenopausal women. EC patients are mainly treated by comprehensive treatment, including surgery, radiotherapy, chemical anticancer drugs, and hormone therapy (2). For early-stage EC patients, the treatments are effective, the risk of recurrence is low, and the prognosis is good. However, for advanced stage EC patients, these treatment options are limited in efficacy and prone to relapse, with a 5-year survival rate of only 10-30% (3, 4). At present, there are no very effective biomarkers to predict the prognosis of patients with EC. Therefore, the development and determination of prognostic markers is imminent.

Alternative splicing (AS) is an important post transcriptional process in which different RNA transcripts are formed by splicing and rearrangement in various ways, resulting in structurally and functionally different protein isoforms, modifying more than 95% of human genes (5–7). In recent years, studies have shown that aberrant AS is closely associated with the occurrence, development, metastasis and drug resistance of various cancers (8–11), including EC. Studies have found that splice factor SF3B1 plays a vital carcinogenic role in the occurrence of EC, and the knockdown of this gene could reduce the proliferation and migration of EC cell lines (12). In addition, by analyzing the whole genome of AS events in EC, some studies have found several candidate splicing factors that may become the therapeutic targets of EC, and can predict the prognosis of patients by constructing gene signatures (13, 14), which further demonstrated the importance of AS events in EC.

AS events are closely related to cancer immunotherapy (15), in which tumor microenvironment (TME) also plays a crucial role. TME is mainly composed of tumor cells, immune and inflammatory cells, tumor-related fibroblasts, stromal tissues, microvessels, and various cytokines and chemokines (16). It is a complex and comprehensive system, closely related to the occurrence, development, and metastasis of tumors (17). There have been many studies that have shown that the level of immune cells in the TME has a crucial impact on the prognosis of patients with cancer and can be a valuable prognostic marker (18–20). The ESTIMATE algorithm is a widely used method to calculate immune and stromal scores in TME, mainly by analyzing their specific gene expression characteristics, so as to promote quantitative analysis of tumor immune and stromal components (21). In EC, previous studies have found that immune and stromal scores are related to the prognosis of patients based on this algorithm, and an immune-related eight-gene signature has been developed to predict the prognosis of EC patients (22).

**Figure 1:**
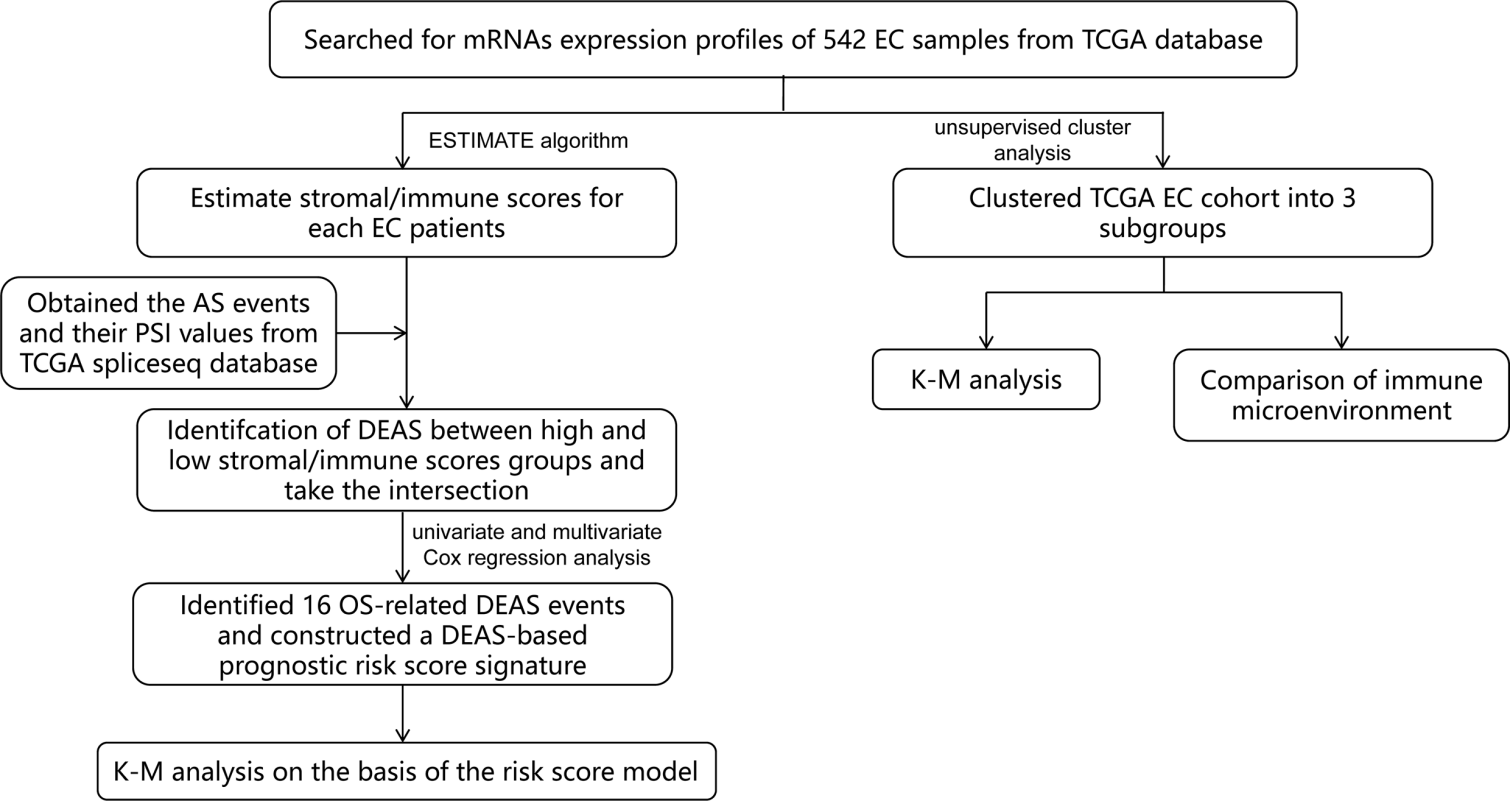
Flow chart of the bioinformatic analysis.

In this study, we combined TME and AS events to provide a more comprehensive analysis of prognostic factors in EC patients, which has not been done before. First of all, by mining public databases and performing an ESTIMATE algorithm, we obtained the immune score and stromal scores of patients with EC. Then, we detected different expressed AS events (DEAS) by comparing the AS events between high and low stromal/immune scores groups. Based on this, we constructed a sixteen-gene signature (**Figure 1**) and found that it was a prognostic indicator independent of other clinicopathological parameters. These findings help us better assess the prognosis of EC patients and provide assistance for clinical diagnosis.

## Method

### Data Collection and Estimation of Stromal and Immune Scores

We extracted the mRNA expression profiles and clinical information of 542 EC patients from The Cancer Genome Atlas (TCGA) database (https://www.cancer.gov/). The clinical information of the patients included age, Neoplasm Histologic Grade, American Joint Committee on Cancer (AJCC) stage, OS and TCGA molecular classification (**Supplementary Table 1**). We also obtained the AS events and their percent-splice-in (PSI) values from TCGA spliceseq database. PSI values (ranging from 0 to 1) were used for quantification and analysis of AS events. In order to ensure the reliability of the AS events we obtained, percentage of samples with PSI values > 75% were included for further study. In addition, an ESTIMATE (Estimation of STromal and Immune cells in MAlignant Tumor tissues using Expression data) algorithm was performed to estimate the stromal score and immune score for each EC sample.

### Analysis of the Relationship Between Stromal/Immune Scores and Prognosis of EC Patients

Based on the median stromal/immune scores, we divided EC patients into high and low stromal/immune scores groups and compared the outcome between these two groups by adopting the Kaplan-Meier (K-M) survival curves and the log-rank test.

### Identification and Analysis of DEAS Events Between High and Low Stromal/Immune Scores Groups

We made statistics on AS events of EC, “UpSetR” package was applied to summarize the intersections between AS events and the corresponding gene intersections, and the results were visualized by UpSet plots. Next, we used “limma” R package to compare AS events with high and low stromal/immune scores groups and defined as DEAS events with a false discovery rate (FDR) of < 0.05, which were plotted by volcano plots and heatmap. Then a Venn diagram was used to identify DEAS events that were up-regulated or down-regulated in both stromal score and immune scores groups. In addition, to further understand the potential functions and enrichment pathways of DEAS events, we conducted Gene Ontology (GO) and Kyoto Encyclopedia of Genes and Genomes (KEGG) pathway enrichment analyses for these DEAS events by the “clusterprofiler” package. GO terms includes biological process (BP), cellular component (CC), and molecular function (MF). The results were displayed by a bubble plot.

### Construction and Analysis of the Prognostic Risk Score Signature Based on the AS Events

To determine events related to patients’ survival in DEAS events, univariate Cox regression analysis was performed to confirm. Then, the LASSO analysis was used to further select the most suitable DEAS events to avoid overfitting and included significant genes into multivariate Cox analysis to construct the prognostic signature based on these DEAS events. The expression value of selected DEAS events and the regression coefficient of multivariate Cox regression analysis were combined linearly to establish the following predictive risk scoring model:

$$\text{risk}\;\text{score}=\sum_{i=0}^{n}\text{PSI}\times {\text{β}}_{\text{i}}(\text{β is}\;\text{the}\;\text{coefficient}\;\text{of}\;\text{the}\;\text{AS}\;\text{events})$$

For the accuracy of the survival analysis, 523 patients with EC were finally included by excluding a patient with a survival time of 0 day in clinical information. Subsequently, 523 EC patients were separated into high- and low-risk groups based on the optimal cutoff value of risk score which was determined by performing X-tile software, and the prognostic significance of the risk score were appraised by K-M survival curve and the log-rank test. Additionally, the time-dependent receiver operating characteristic (ROC) curve and area under the curve(AUC) value were adopted to assess the discrimination of the prognostic model.

### Identification the Independence of Prognostic Risk Score Model

To further validate whether the prognostic risk score was independent of the clinicopathological parameters of EC patients including age, AJCC stage, grade, race, margin status, surgical approach, and TCGA molecular classification, the univariate and multivariate Cox regression analysis were conducted.

Moreover, the TCGA molecular classification can be used to predict the prognosis of patients and guide the formulation of clinical treatment plan, providing a new typing choice for the precise treatment of EC (23). Thus, stratified analysis was applied to further validate whether the risk score was independent of TCGA molecular classification including microsatellite instability (MSI), POLE, Copy-number low (CN-HIGH), and Copy-number high (CN-LOW). K-M survival curves were drawn to see the difference of OS in EC patients between the high- and low-risk groups.

### Identification and Analysis the Immune Microenvironment by Consensus Clustering

Additionally, the ConsensusClusterPlus package was performed to do hierarchical consensus clustering analysis on the TCGA EC cohort, and the 524 EC patients was classified into several clusters. Next, the K-M survival curve and the log-rank test were carried out to analyze the difference of prognostics among these subgroups. For each EC sample, the single-sample gene-set enrichment analysis (ssGSEA) was performed to quantify the enrichment levels of the 29 immune signatures including immune cell types and functions, human leukocyte antigen (HLA), tumor-infiltrating lymphocytes (TILs) and so on. Then, we compared the ESTIMATE scores and ssGSEA scores of each subgroup to analyze the relationship between prognosis and immune microenvironment.

### Construction of Potential SF-AS Regulatory Network

Splicing factors (SF) are protein factors involved in the splicing process of RNA precursors, which are closely related to the development and treatment of cancer (24, 25). Thus, we downloaded the SFs data from the SpliceAid2 database and then analyzed the correlation between the expression level of SFs and PSI values of OS-associated AS events by Spearman correlation analysis. The absolute value of correlation coefficient > 0.5 and P < 0.001 were considered statistically significant. Finally, Cytoscape software was used to visualize the potential SF-AS regulatory network.

### Statistical Analysis

All of the statistical analyses were performed using R version 3.6.1, GraphPad Prism 7.0 software and SPSS 16.0. And P < 0.05 was considered significant.

## Results

### The Relationship Between Stromal/Immune Scores and Prognosis of EC Patients

**Figure 2:**
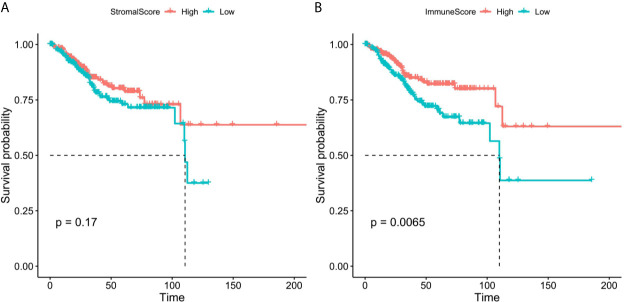
The K-M survival curves of high and low stromal/immune scores groups. **(A)** Stromal scores. **(B)** Immune scores.

We obtained the mRNA expression profile of 542 patients with EC from TCGA database, and the stromal/immune scores of these patients were obtained by estimate algorithm (**Supplementary Table 2**). Then we divided the 542 patients into high and low stromal/immune scores groups based on the medium of stromal scores and immune scores, and the differences of prognosis of EC patients between high- and low- groups were compared. As a result, the K-M curves showed that low immune scores were notably related to poorer survival of patients with EC (**Figure 2A**), however, there was no significant correlation between stromal scores and prognosis of patients with EC (**Figure 2B**).

### DEAS Events Between High and Low Stromal/Immune Scores Groups

**Figure 3:**
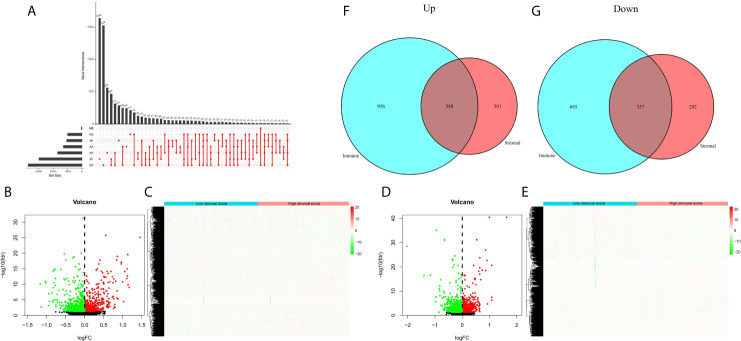
DEAS events between high and low stromal/immune scores groups. **(A)** The UpSet plot of intersections between AS events and the corresponding gene intersections. The volcano plots **(B, D)** and heatmaps **(C, E)** of DEAS events between the high and low stromal/immune scores groups. The up-regulated **(F)** or down-regulated **(G)** DEAS events in both stromal score and immune scores groups by Venn diagram.

Next, we sorted out AS events in patients with EC, and found that ES (Exon Skip) was the most frequent AS event, followed by AT (Alternate Terminator), and ME (Mutually Exclusive Exons) was the least frequent AS event. The detailed information of intersections between AS events and the corresponding gene intersections was visualized in the UpSet plot (**Figure 3A**). Then, we obtained the DEAS events by comparing the high and low stromal/immune scores groups, which was showing in the volcano plots and heatmaps (**Figures 3B–E**). As a result, in immune groups, we obtained a total of 1304 up-regulated DEAS events and 1302 down-regulated DEAS events; in the stromal groups, we obtained 649 up-regulated DEAS events and 629 down-regulated DEAS events. By applying the Venn diagram software, finally, we detected DEAS events that were up-regulated or down-regulated in both the stromal score and immune score groups, including 348 up-regulated and 337 down-regulated DEAS events (**Figures 3F, G**).

**Figure 4:**
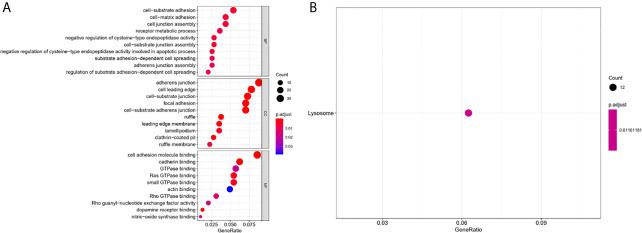
GO and KEGG pathway enrichment analysis of DEAS events. **(A)** GO analysis. **(B)** KEGG pathway enrichment analysis.

To further comprehend the biological function and significant pathways of these DEAS events, the GO analysis and KEGG pathway enrichment analysis were carried out on the genes corresponding to DEAS events. In the BP category, cell–substrate adhesion and cell–matrix adhesion were most enriched; in the CC category, cell leading edge and adherens junction were the main enriched GO terms; in the MF category, the primary function of these genes were cell adhesion molecule binding and cadherin binding (**Figure 4A**). As for KEGG pathway enrichment analysis, the significant pathway was lysosome (**Figure 4B**).

### Construction the DEAS-Based Prognostic Risk Score Model

**Figure 5:**
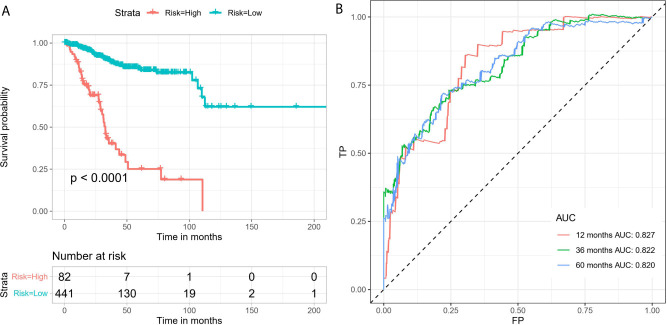
The 16 OS-related DEAS events signatures associated with risk score predicts EC patients’ OS. **(A)** K-M survival curve to test the predictive effect of the gene signature. **(B)** ROC curve analysis to evaluate the sensitivity and specificity of the gene signature.

By conducting univariate Cox regression analysis for the above DEAS events that were both up-regulated or down-regulated, we screened out 148 OS-related DEAS events with P <0.05 (**Supplementary Table 3**). Next, following the LASSO analysis and multivariate Cox analysis we obtained 16 OS-related DEAS events (ANAPC11|44217|ES, CCDC180|86996|AT, SH3BP2|68594|AP, SLC3A2|16462|AP, RAF1|63446|ES, TSC22D3|89836|AP, NCOA4|11539|AD, DPH6|29893|AT, SPEG|57696|AT, CYB561A3|16165|RI, EVL|29239|AP, SCRIB|98107|ES, NEDD9|75338|AP, AHI1|77886|AT, TRAPPC6A|50410|ES, CREM|11230|AP) and their coefficients (**Table 1**). According to the formula mentioned in the method, we calculated the risk score of each EC patient, and based on the optimal cutoff value as the boundary value, we divided the patients into a high and a low-risk group. Then, by performing K-M survival analysis, we found that the low-risk group had significantly better OS than the high-risk group with P < 0.0001 (**Figure 5A**). And the AUC values for 1-, 3-, and 5-year OS were 0.827, 0.822, and 0.820, respectively (**Figure 5B**), indicating that the DEAS-based prognostic risk score signature has a good ability to predict the OS of EC patients.

**Table 1:** The information of 16 OS-related DEAS events by multivariate Cox analysis.

ID	coef	HR	95%CI	pvalue
ANAPC11|44217|ES	3.21	24.71	1.75-349.50	0.018
CCDC180|86996|AT	-0.99	0.37	0.10-1.43	0.149
SH3BP2|68594|AP	-1.65	0.19	0.04-0.82	0.025
SLC3A2|16462|AP	3.91	49.80	4.35-569.97	0.002
RAF1|63446|ES	5.96	388.02	1.58-95252.57	0.034
TSC22D3|89836|AP	1.93	6.91	0.78-60.92	0.082
NCOA4|11539|AD	-8.51	0.00	7.42E-09-5.49	0.102
DPH6|29893|AT	-5.89	0.00	6.67E-05-0.12	0.002
SPEG|57696|AT	-1.93	0.14	0.01-1.82	0.135
CYB561A3|16165|RI	1.23	3.43	0.87-13.53	0.079
EVL|29239|AP	1.54	4.66	1.82-11.95	0.001
SCRIB|98107|ES	-1.56	0.21	0.05-0.85	0.029
NEDD9|75338|AP	-2.98	0.05	0.00-1.67	0.094
AHI1|77886|AT	1.25	3.49	0.65-18.63	0.144
TRAPPC6A|50410|ES	1.34	3.81	0.59-24.65	0.160
CREM|11230|AP	2.48	11.95	2.71-52.63	0.001

### The DEAS-Based Risk Score Was an Independent Prognostic Indicator

In univariate Cox regression analysis we found that risk score, age, AJCC stage, grade, margin status, and TCGA molecular classification were significantly associated with OS (**Table 2**). Next, we further performed the multivariate Cox regression analysis to avoid the collinearity between variables. As a result, we found risk score, grade, and AJCC stage were independently related to EC patients’ OS (**Table 3**). In general, the risk score was an independent prognostic indicator.

**Table 2:** Univariate Cox regression analyses for identifying clinicopathological parameters related to EC patients’ OS.

Clinical feature	Univariate analysis		
	HR	95%CI of HR	P value
Risk High	7.16	4.67-10.99	<0.001
Age, y	1.03	1.01-1.05	0.002
Race			
Black	1 (reference)		
White	0.95	0.56-1.60	0.835
Other	0.65	0.24-1.74	0.388
AJCC stage			
I	1 (reference)		
II	1.91	0.88-4.15	0.104
III	3.27	1.99-5.37	<0.001
IV	9.30	5.12-16.90	<0.001
Grade			
Low	1 (reference)		
High	3.19	1.85-5.49	<0.001
Margin_status			
R0	1 (reference)		
R1	1.95	0.78-4.91	0.156
R2	6.62	3.43-12.78	<0.001
Surgical_approach			
Minimally Invasive	1 (reference)		
Open	0.78	0.50-1.21	0.274
TCGA molecular classification			
CN_HIGH	1 (reference)		
CN_LOW	0.26	0.14-0.50	<0.001
MSI	0.39	0.23-0.67	<0.001
POLE	0.09	0.02-0.38	<0.001

**Table 3:** Multivariate Cox regression analyses for identifying clinicopathological parameters related to EC patients’ OS.

Clinical feature	Multivariate analysis		
	HR	95%CI of HR HR	P value
Risk High	4.40	2.32-8.35	<0.001
Age, y	1.02	0.99-1.05	0.223
AJCC stage			
I	1 (reference)		
II	1.29	0.37-4.44	0.687
III	4.11	2.11-8.00	<0.001
IV	9.44	2.76-32.28	<0.001
Grade			
Low	1 (reference)		
High	2.73	1.22-6.11	0.014
Margin_status			
R0	1 (reference)		
R1	1.05	0.36-3.10	0.929
R2	0.82	0.28-2.37	0.714
TCGA molecular classification			
CN_HIGH	1 (reference)		
CN_LOW	2.01	0.84-4.82	0.118
MSI	1.89	0.84-4.25	0.123
POLE	0.31	0.07-1.41	0.129

**Figure 6:**
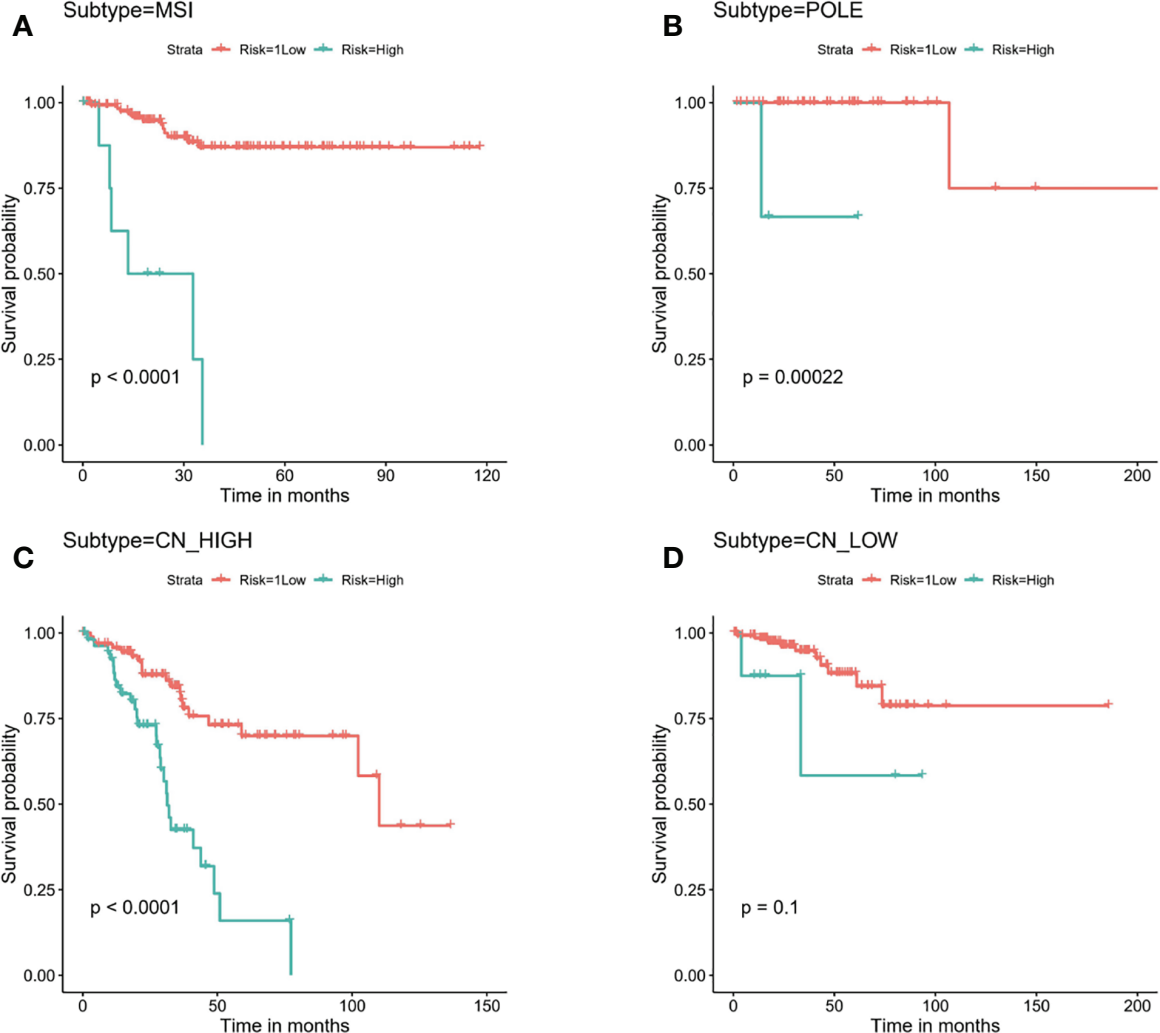
Stratified analysis for the prognostic risk model in the different subgroups according to TCGA molecular classifications. **(A)** MSI, **(B)** POLE, **(C)** CN-HIGH, **(D)** CN-LOW.

Next, stratified analysis was performed to further validate whether the prognostic risk model can be used as an independent prognostic factor in the different subgroups according to TCGA classifications. As a result, we found that the OS of the low-risk group was obviously better than the high-risk group among MSI, POLE, and CN-HIGH subgroups, and their P values were < 0.0001, 0.00022, and < 0.0001, respectively (**Figures 6A–C**). In the CN-LOW subgroup, we can also see the trend of poor prognosis in the high-risk group, although the P value is only 0.1 (**Figure 6D**).

### The Immune Microenvironment Was Closely Related to the Prognosis of EC Patients

**Figure 7:**
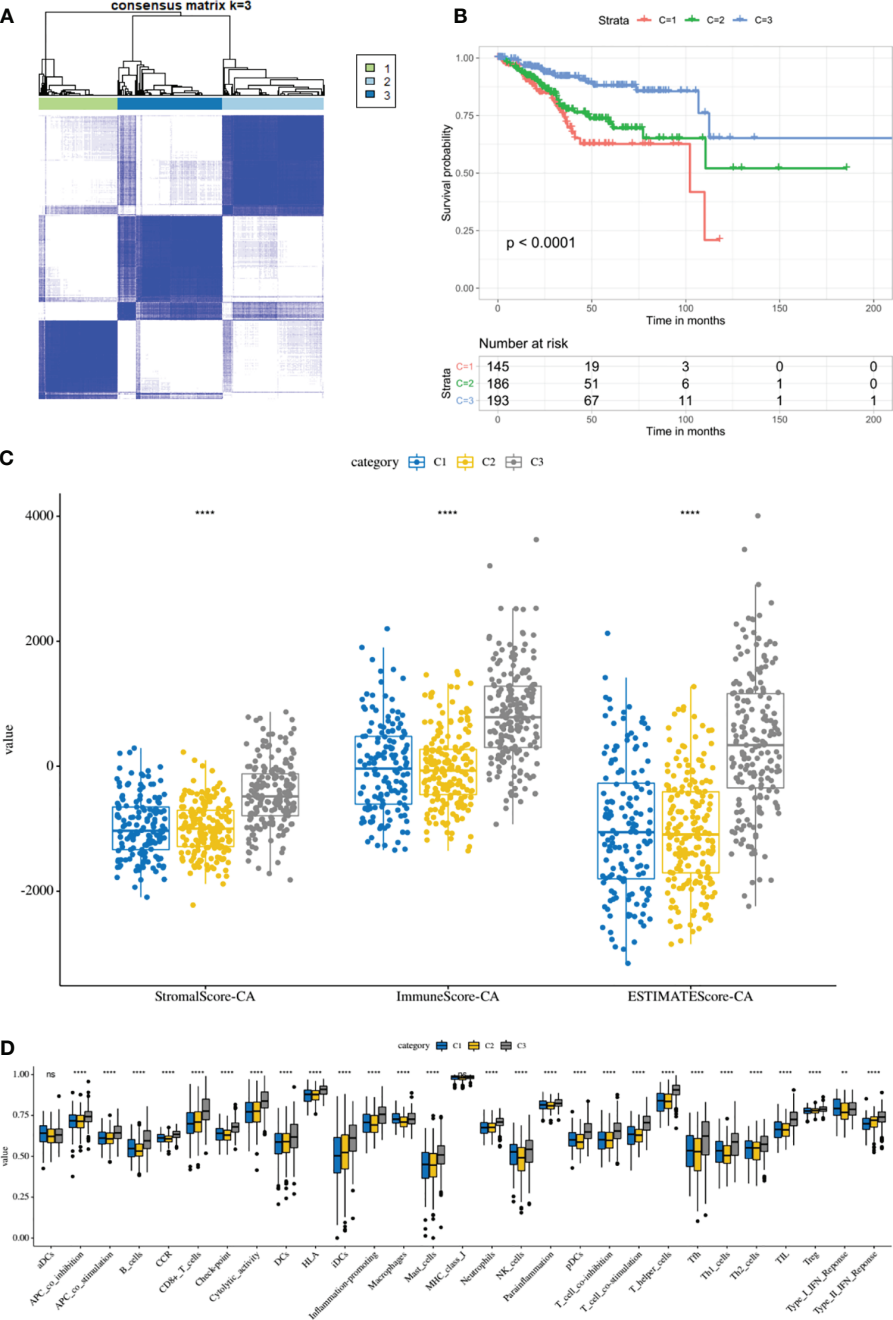
The immune microenvironment was closely related to the prognosis of EC patients. **(A)** TCGA EC cohort was clustered into three subgroups by unsupervised cluster analysis. **(B)** K-M survival curves of three clusters. **(C)** The comparison of stromal scores, immune scores, and ESTIMATE scores between three clusters. **(D)** Box plots for comparison of immune cell infiltration between three clusters. ****, P < 0.0001; NS, not significant.

By performing unsupervised cluster analysis, we clustered the TCGA EC cohort into 3 subgroups (**Figure 7A**) (Cluster 1: 145 samples, Cluster 2: 186 samples, Cluster 3: 193 samples). From the K-M survival curve we can see that the prognosis of C3 was the best, followed by C2 and C1 (**Figure 7B**). As for the immune microenvironment, the stromal scores, immune scores, and ESTIMATE scores were all highest in C3 (**Figure 7C**). In addition, we compared the enrichment levels of the 29 immune signatures in these three subgroups, almost all immune cell infiltration levels were highest in C3 (**Figure 7D**). In general, the immune microenvironment was closely related to the prognosis of EC patients, the higher the level of immune infiltration, the better the prognosis of EC patients.

### Potential Regulatory Network Between SFs and AS Events

**Figure 8:**
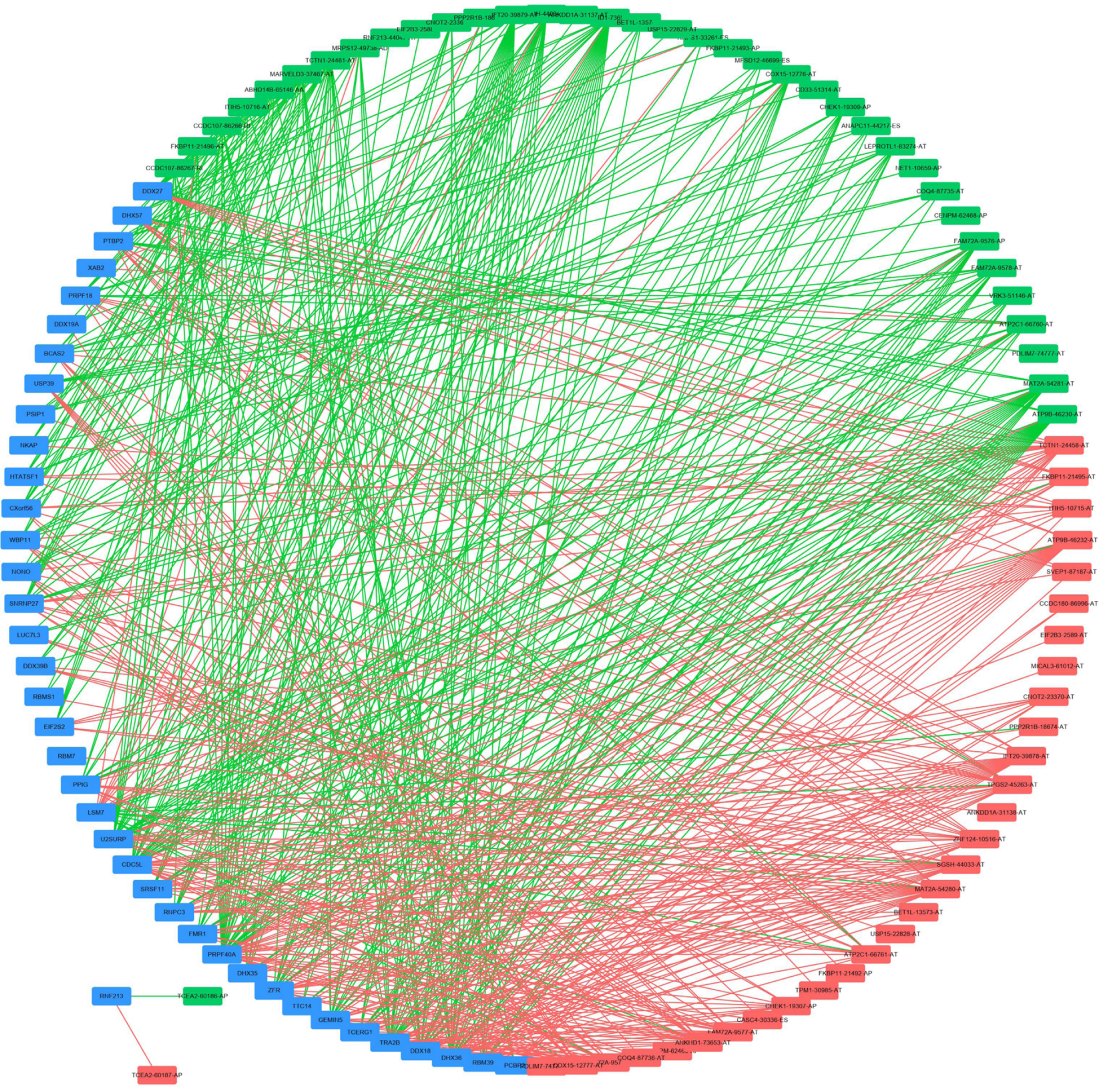
Regulatory network between SFs and AS events. Thirty-nine SFs (blue) were significantly related to 68 survival-associated AS events consisting of 37 adverse AS events (green) and 31 favorable AS events (red). The majority of adverse AS events were positively correlated with SF expression (green lines) and the most favorable AS events were negatively correlated with SF expression (red lines).

To explore the underlying regulatory network between SFs and AS events in EC patients, we first downloaded 390 SFs data from the SpliceAid2 database. By Spearman correlation analysis, we screened out 39 SFs (blue) which were significantly related to 68 survival-associated AS events consisted of 37 adverse AS events (green) and 31 favorable AS events (red) (**Figure 8**). Furthermore, the majority adverse AS events were positively correlated with SF expression(green lines) and the most favorable AS events were negatively correlated with SF expression (red lines) (**Figure 8**).

## Discussion

In recent years, the emergence of immunotherapy has completely changed the situation of traditional cancer treatment and created a new era of tumor immunotherapy, and it also plays a decisive role in the treatment of endometrial cancer. For example, the results of the keynote-028 study showed that pembrolizumab had sustained antitumor activity with ORR 13% in PD-L1 positive patients with advanced EC (26). In addition, EC patients with mismatch repair defect(MMRD) also had a good effect on immunotherapy, in a phase II study it was found that avelumab exhibited promising activity on this kind of patient, and it was not related to the expression of PD-L1(NCT02912572) (27); another phase I found that the ORR of these patients could reach 42.3% after dostarimab treatment(NCT02715284) (28). Although immunotherapy has brought new hope to patients with EC, there are still many patients who cannot benefit from it. Therefore, it is still necessary to explore immune related prognostic markers and further develop new treatment strategies to improve the prognosis of EC patients. In this study, we first identified immune related DEAS events to construct a risk score model to predict the outcome of EC patients and achieved favorable prediction results.

First, we downloaded the mRNA expression profile of 542 patients with EC from TCGA database and performed ESTIMATE algorithm to estimate the stromal/immune scores of each sample. Then, the DEAS events were obtained by comparing the high and low stromal/immune scores group and 16 OS-related DEAS events was obtained through univariate and multivariate Cox regression analysis (P < 0.05). Afterward, the DEAS-based prognostic risk score signature was constructed, and we can see that prognostic of EC patients in high-risk group was significantly poorer than low-risk group with P < 0.0001. Furthermore, we found the prognostic risk score was independent of the clinical information and TCGA molecular classification of EC patients. And based on the stratified analysis, we found that in MSI, POLE, and CN-HIGH subgroups, patients in the high-risk group had significantly poor prognosis, and there was the same trend in CN-LOW subgroup. And in the CN-LOW subgroup, we believe that the poor P value might be due to the small number of patients. Last but not least, we clustered EC patients into three subgroups and found that EC patients will have better survival when they have higher levels of immune infiltration and ESTIMATE scores.

According to GO and KEGG enrichment analysis, we found that these DEAS events, including cell–substrate adhesion, adherens junction, cell adhesion molecule binding, lysosome, and so on, were all closely related to the initiation, growth, and progression of tumors. Cell–subject adhesion is an important regulator of cell migration, differentiation and tissue integrity, and affects the invasion and metastasis of malignant cells (29); Adherens junction is closely related to the invasion and migration of tumor cells (30), for example, E-cadherin is one of the main components of adhesions junctions, which is an invasion and tumor suppressor, and the loss of E-cadherin is associated with poor prognosis of various cancers, including ovarian cancer (31), prostate cancer (32), head and neck cancer (33), and so on; Lysosomes are the center of cell degradation, and their abnormalities can lead to uncontrolled cell growth, which can contribute to the progression of cancer (34).

Furthermore, in our research, among 16 OS-related DEAS events, there were nine risk factors(ANAPC11|44217|ES, SLC3A2|16462|AP, RAF1|63446|ES, TSC22D3|89836|AP, CYB561A3|16165|RI, EVL|29239|AP, AHI1|77886|AT, TRAPPC6A|50410|ES, CREM|11230|AP) and seven protective factors (CCDC180|86996|AT, SH3BP2|68594|AP, NCOA4|11539|AD, DPH6|29893|AT, SPEG|57696|AT, SCRIB|98107|ES, NEDD9|75338|AP). These genes were involved in the occurrence and development of various cancers to varying degrees and affect the prognosis of cancer patients. For example, Anaphase promoting complex subunit 11 (ANAPC11) mainly plays a role in the regulation of the cell cycle. Some studies have found that in colon cancer its overexpression is related to chromosome instability, lymphovascular invasion, and residual tumor, as well as poor relapse free survival rate and poor overall survival rate, which may be a potential predictor of metastatic colon cancer (35). In addition, its overexpression was found to be associated with liver cancer cell migration, which may promote the progression of liver cancer (36). Some studies have found that SLC3A2 has a carcinogenic effect in many kinds of cancers, including oropharyngeal cancer (37), head and neck squamous cell carcinomas (HNSCC) (38, 39), and renal cancer cell (40), which contributes to inferior prognosis of cancer patients, and may act as a predictive marker. RAF1 is a kind of gene known as an oncogene, which is a part of the RAS/MAPK pathway, and is closely related to the progression of a variety of tumors and can be a potential key target of cancer therapy (41–44). TSC22D3 is a glucocorticoid-inducible transcriptional regulator and also an immunosuppressive transcription factor, the activation of this gene will lead to immunosuppressive effect and the failure of the anti-tumor immunotherapy (45–47). Ena/VASP-like(EVL) encodes actin related proteins and plays an important role in regulating actin cytoskeleton (48, 49). Studies have found that EVL may be related to the invasion and metastasis of breast cancer, and its up-regulation is positively correlated with the clinical staging of breast cancer (50). Abelson helper integration site-1 (AHI1) is an oncogene and has an oncogenic effect on the development of human leukemia (51–53). These nine risk factors were nearly all closely associated with the prognosis of cancer patients, though there is no related research on EC, which needs further studies in the future. As for the protective factors, Scribble(Scrib) is a gene related to cell polarization and proliferation regulation (54). It was first found in the genetic analysis of drosophilas, Scribble loss would lead to overproliferation, migration, and invasion of epithelial cells, which is also known as neoplastic tumor suppressor gene (nTSG). Moreover, it is also closely related to human cancers, and its down-regulation will promote the progress of tumors, including breast cancer (55), endometrial cancer, prostate cancer (56), and so on. SH3 domain-binding protein 2 (SH3BP2) is a pathogenic gene of Cherubism and some studies have suggested that its protein was helpful to regulate the signaling pathway of B cells and macrophages in the immune system. However, its role in cancer is still unclear, several studies have found that SH3BP2 was a protective factor (57–59) and may be a tumor suppressor gene in bladder cancer (60). In our study, these two genes were also found to be protective factors, which is consistent with previous studies.

In addition, we have divided the TCGA EC cohort into three subgroups by unsupervised cluster analysis. We found that the patients with the best prognosis not only had the highest stromal score, immune score, and ESTIMATE score, but also had the highest level of immune cell infiltration. Previous studies have demonstrated that immune cells are indeed an important prognostic factor in patients with EC (61). For example, patients with uterine tumors will benefit from the treatment if NK cells are present in uterine tumors (62). And Svetlana et al. have found that the higher the tumor-infiltrating CD8+ T cells in EC patients, the higher the overall survival rate would be, which may be a very reliable independent prognostic factor (63). Jones et al. have found that cytotoxic lymphocyte immune signature and T-cell trafficking signature were closely related to the prognosis of female malignancies such as EC, further confirming that immune cell infiltration in the tumor microenvironment affected survival and treatment outcomes in patients with EC (64).

It is undeniable that there are still some defects in our research. First of all, it would be better if there was an independent external validation cohort to validate our DEAS-based prognostic risk score signature. Second, our study was based entirely on bioinformatics analysis and would have been better if some experimental validation had been carried out.

In conclusion, we first used the ESTIMATE algorithm to divide EC patients into high and low immune/stromal score groups and compared them to obtain 16 OS-related DEAS events. Based on this, we constructed a gene signature to predict the outcome of EC patients, and found that the patients in the high-risk group had significantly worse OS. Additionally, we further confirmed that the prognosis of patients was significantly related to the immune microenvironment by unsupervised cluster analysis.

## Conclusion

In general, the prognosis of patients with EC is significantly related to immunity. There are differences in AS events among patients with different ESTIMATE scores, and the gene signature constructed based on these DEAS events can predict the prognosis of EC patients, which provides profound thinking and new insights for clinical patients with EC.

## Data Availability Statement

The original contributions presented in the study are included in the article/**Supplementary Material**. Further inquiries can be directed to the corresponding authors.

## Author Contributions

LC conceived and designed the study with SY. XL and CL drafted the manuscript and analyzed the data. JL and YS handled the picture and article format. SW and MW reviewed the data. All authors contributed to the article and approved the submitted version.

## Conflict of Interest

The authors declare that the research was conducted in the absence of any commercial or financial relationships that could be construed as a potential conflict of interest.

## Supplementary Material

Supplementary Table 1 The clinical information of the EC patients.

Supplementary Table 2 The stromal/immune scores of EC patients from TCGA database.

Supplementary Table 3 The information of 148 OS-related DEAS events by univariate Cox regression analysis.
